# The effect of changing the sequence of cuff inflation and device fixation with the LMA-Supreme® on device position, ventilatory complications, and airway morbidity: a clinical and fiberscopic study

**DOI:** 10.1186/1471-2253-14-2

**Published:** 2014-01-04

**Authors:** Ingo Bergmann, Thomas Allen Crozier, Markus Roessler, Hanna Schotola, Ashham Mansur, Benedikt Büttner, José Maria Hinz, Martin Bauer

**Affiliations:** 1 Department of Anaesthesiology, Emergency and Intensive Care Medicine, University of Göttingen Medical School, Robert-Koch Str. 40, 37075 Göttingen, Germany

**Keywords:** Supraglottic airway, Insertion sequence, Malposition, Endoscopic evaluation, Glottic narrowing, Ventilatory impairment, Airway morbidity

## Abstract

**Background:**

The conventional sequence when using supraglottic airway devices is insertion, cuff inflation and fixation. Our hypothesis was that a tighter fit of the cuff and tip could be achieved with a consequently lower incidence of air leak, better separation of gastrointestinal and respiratory tracts and less airway morbidity if the device were first affixed and the cuff then inflated.

**Methods:**

Our clinical review board approved the study (public registry number DRKS00003174). An LMA Supreme® was inserted into 184 patients undergoing lower limb arthroscopy in propofol-remifentanil anaesthesia who were randomly assigned to either the control (inflation then fixation; n = 92) or study group (fixation then inflation; n = 92). The cuff was inflated to 60 cmH_2_O. The patients’ lungs were ventilated in pressure-controlled mode with 5 cmH_2_O PEEP, Pmax to give 6 ml kg^-1^ tidal volume, and respiratory rate adjusted to end-tidal CO_2_ of 4.8 and 5.6 kPa. Correct cuff and tip position were determined by leak detection, capnometry trace, oropharyngeal leak pressure, suprasternal notch test, and lube-tube test. Bowl and cuff position and the presence of glottic narrowing were assessed by fiberscopic examination. Postoperative dysphagia, hoarseness and sore throat were assessed with a questionnaire. Ventilatory impairment was defined as a tidal volume < 6 ml kg^-1^ with Pmax at oropharyngeal leak pressure, glottic narrowing was defined as an angle between the vocal cords under 16 degrees.

**Results:**

The incidence of incorrect device position (18% vs. 21%), failed ventilation (10% vs. 9%), leak pressure (24.8 vs. 25.2 cmH_2_O, p = 0.63), failed lube-tube test (16.3% vs. 17.6%) and glottic narrowing (19.3% vs. 14.1%, p = 0.35) was similar in both groups (control vs. study, resp.). When glottic narrowing occurred, it was more frequently associated with ventilatory impairment in the control group (77% vs. 39%; p = 0.04). Airway morbidity was more common in the control group (33% vs. 19%; p < 0.05).

**Conclusions:**

Altering the sequence of cuff inflation and device fixation does not affect device position, oropharyngeal leak pressures or separation of gastrointestinal and respiratory tracts. It reduces the incidence of glottic narrowing with impaired ventilation and also perioperative airway morbidity.

## Background

Supraglottic airways are now widely used in clinical anaesthesia, and although the limitations of the original model have been addressed and a wide variety of improved models are available, the incidence of perioperative complications associated with their use is still between 8% and 19% [1-5]. These complications range from serious, e.g. the inability to adequately ventilate the patient’s lungs, potentially serious, as an insufficient separation of the gastrointestinal and respiratory tracts to minor complaints such as postoperative dysphagia, hoarseness and sore throat. Narrowing of the glottis related to the device has been reported in about 10% of the patients [1,6,7].

Based on theoretical considerations, we hypothesised that the sequence of cuff inflation and tube fixation might influence both the seating of the cuff in the surrounding anatomical structures as well as the factors causing airway morbidity. To the best of our knowledge, in all published studies, the device was inserted, the cuff inflated and the device then fastened to the patient with tape. The increasing size of the cuff during inflation might force it out of an initially correct position causing an air leak. Friction between the cuff and the soft tissues of the pharynx and hypopharynx can cause airway morbidity. The concept was that by first fastening the device, the cuff would have to expand downward and force the tip into a tighter fit in the upper oesophageal sphincter.

We tested the hypothesis that securing the device first and then inflating the cuff would reduce the incidence of an inadequate seal and of airway morbidity. We used a single model of supraglottic airway device to minimise confounding factors. We chose one with an integrated drainage channel (LMA-Supreme®) that allows one to assess tip position with simple clinical tests.

## Methods

This prospective, randomised study was approved by our institutional clinical study review board (Ethikkommission der Universitätsmedizin Göttingen) and registered in a publicly available registry under the number DRKS00003174. It was conducted in the period from June 2011 to May 2012. The participants were 18 to 75 year-old, ASA I-III patients scheduled for elective lower-limb arthroscopy in the supine position who had given written informed consent. Exclusion criteria were a history of radiation therapy or surgery of the neck or hypopharynx, a mouth opening less than 3 cm, a known or expected difficult airway, gastric reflux or a BMI over 35. Immediately before induction of anaesthesia, the patients were allotted to one of the two study groups using the computer-generated randomisation list described below. The groups were “conventional sequence”, i.e. cuff inflation then device fixation, and “study sequence”, i.e. device fixation then cuff inflation.

The primary endpoints were oropharygeal leak pressure and sufficient separation of gastrointestinal and respiratory tracts. Secondary endpoints were fibreoptically assessed *in situ* position, occurrence of glottic narrowing, impaired ventilation, evidence of airway morbidity.

### Anaesthesia

On the patients’ arrival in the operating theatre, we inserted a peripheral venous cannula, established monitoring of ECG, non-invasive blood pressure, peripheral oxygen saturation and depth of anaesthesia (Entropy Sensor®, GE Healthcare), and gave intravenous midazolam for anxiolysis (1–3 mg titrated to effect).

After a ten-minute rest period, we recorded the baseline values of blood pressure, heart rate, and state and response entropy (SE; RE) and then induced anaesthesia with remifentanil (bolus injection 1 μg.kg^-1^, continuous infusion at 0.2 μg.kg^-1^.min^-1^) and propofol. Propofol was administered initially at a rate of 1 mg.kg^-1^.min^-1^ until the state entropy value dropped below 60. We then adjusted the infusion rate to keep the SE value between 40 and 60. After the patient stopped breathing we manually ventilated the lungs by bag and mask.

After confirming loss of muscle tone by forced jaw thrust, one investigator (IB) inserted the lubricated (Endosgel®, Farco-Pharma, Cologne, Germany) LMA-Supreme® device (LMA-S) according to the manufacturer’s instructions. We initially used a size 4 LMA-S in all patients, since this is the correct size for the majority of our patient population and because the manufacturer recommends it as the first choice. The distance between the lips and the fixation tab of the LMA-S was measured and recorded. According to the manufacturer’s criteria the mask size is correct if this distance is between 0 and 2.5 cm. The following steps were then performed according to the study protocol. In the control group, we inflated the cuff to 60 cm H_2_O and secured the tube with tape following the manufacturer’s instructions. In the study group, we taped the tube of the device in the same manner and then inflated the cuff to. The lip-to-fixation tab distance was measured again. Apart from the sequence of blocking the cuff and securing the airway device, patient treatment was identical in both groups (see flow chart, Table 1). In both groups we checked cuff inflation pressure frequently and kept it at 60 cmH_2_O.

**Table 1:** Flow chart of study

**Control group**	**Study group**
Induction	Induction
Insertion of LMA-S	Insertion of LMA-S
***Cuff inflation***	***Fixation***
***Fixation***	***Cuff inflation***
Ventilation	Ventilation
Check seating	Check seating
If necessary, correct and check again	If necessary, correct and check again
Ventilate	Ventilate
Fiberscopic assessment of position	Fiberscopic assessment of position
End of surgery	End of surgery
Evaluate airway morbidity	Evaluate airway morbidity

Two investigators who were blinded to the patients’ group allocations (BB and HS) performed the following measures. They connected the LMA to the respirator in pressure-controlled mode with a PEEP of 5 cmH_2_O and an initial maximal inspiratory pressure of 15 cmH_2_O. The pressure was increased step-wise until a tidal volume of 6 ml.kg^-1^ was achieved. We auscultated the anterolateral neck and the mouth to detect any audible leakage. The position of the device was accepted and graded as “adequate oropharyngeal seal” if there was no audible leak at this tidal volume, and if the capnography trace showed a normal plateau phase. If these criteria were not fulfilled, we allowed two attempts to improve the seating. The permitted corrective measures were adjusting the mask’s position without removing it, removing and reinserting the same device or exchanging it for one of a different size with the restriction that size change was never the first measure. The necessity of corrective measures was documented. If the functional seating criteria were not fulfilled after the second attempt, the LMA-S was removed, the trachea was intubated, and the patient was graded as “failed” and excluded from further evaluation. Oropharyngeal leak pressure was determined by slowly increasing the airway pressure with a constant fresh gas flow into the circuit in the closed manual mode until an audible leak was detected from the mouth and/or by ausculation over the larynx [8].

We assessed the separation of the gastrointestinal and respiratory tracts with two further tests. In the suprasternal notch test a small amount of lubrication gel is introduced into the drainage channel, and pressure on the jugulum forces the gel upwards if the tip of the device is correctly positioned in the upper oesophageal sphincter. In the “lube-tube test” the gel in the drainage channel should show an oscillatory movement during ventilation but not leave the tube when the tip is correctly positioned and the oesophagus and the trachea are adequately separated [9]. Failure of these tests gave a rating of “incorrect tip position”. In a final test, a nasogastric tube was inserted through the drainage channel; insertion was not possible if the tip of the device was bent or not in the entrance to the oesophagus. If any of these tests failed, the corrective measures described above were performed.

**Figure 1:**
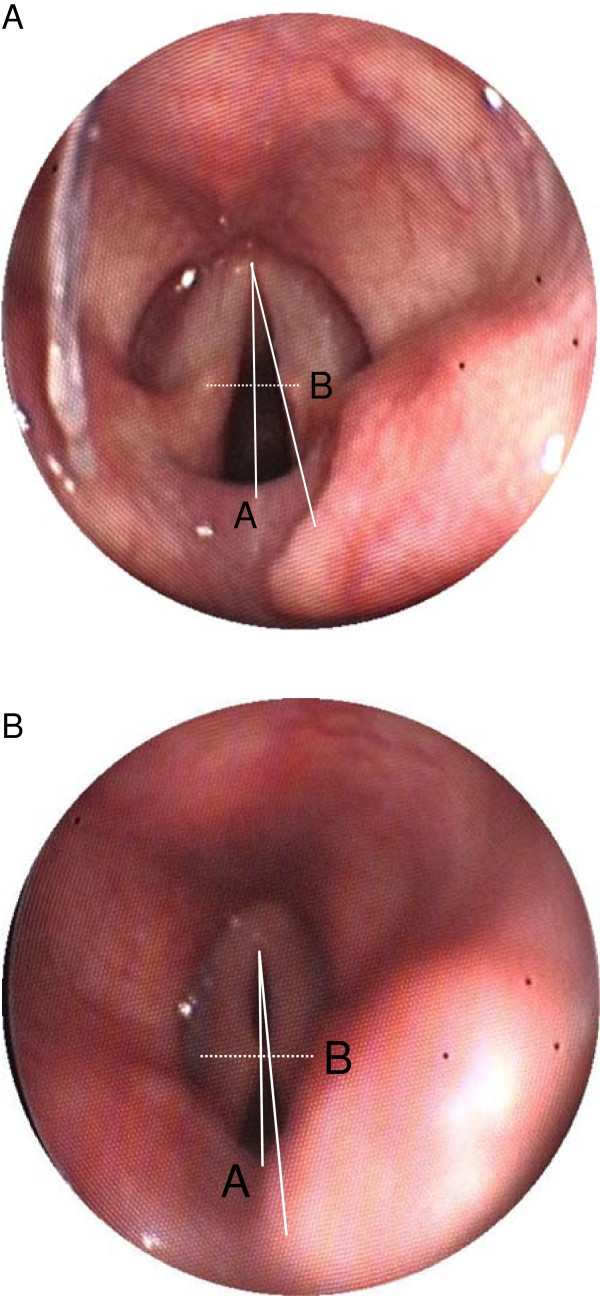
**The opening angle of the glottis was measured on a hardcopy of the photograph taken during endoscopic inspection.** A sagittal line was drawn connecting the anterior commissure and the interarytenoid notch (line A). Line B was drawn bisecting line A at a right angle. A tangential line was drawn from the intersection of the sagittal line with the anterior commissure through the intersection of line B with the vocal cord. The angle between lines A and B was measured and doubled to give the opening angle. This method permitted the opening angle to be determined when only one vocal cord was visible. **A**. Glottis with an opening angle of 26° - ventilation not impaired. **B**. Glottis with an opening angle of 9° - ventilation impaired.

After these tests had demonstrated that the position of the tip was correct, we inserted a 3.5 mm diameter fiberscope (Karl Storz GmbH Endoskope; Tuttlingen) through the ventilation tube in apnoea, with which we viewed and photographed the position of the bowl and tip, and the anatomy of hypopharyngeal structures. The correct position of the device’s tip is posterior to the arytenoid cartilage with the epiglottis visible and not reflected over the glottic opening. The vocal cords should be visible with no narrowing of the glottic opening [1]. We defined relevant glottic narrowing endoscopically as an angle of 16 degrees or less between the vocal cords or a closure of the ventral glottic opening (Figure 1). This cut-off angle was detected by two investigators (BB and IB) and is based on an analysis of unpublished data.

For surgery the respirator was in pressure-control mode with the following settings: PEEP 5 cmH_2_O, Pmax sufficient to give a tidal volume of at least 6 ml.kg body weight^-1^ and a respiratory rate adjusted to keep end-tidal CO_2_ between 4.8 and 5.6 kPa. Endoscopy was repeated whenever there was an unexpected increase in airway pressure or decrease in tidal volume to determine a possible cause. We defined ventilatory impairment as a reduction of tidal volume to less than 6 ml.kg^-1^ and/or a required Pmax greater than the oropharyngeal leak pressure. If glottic narrowing was identified as a possible cause, we deepened anaesthesia with bolus injections of propofol (50 mg) and remifentanil (50 μg) and then reassessed the glottis. If this did not improve the glottic narrowing, the LMA-S was removed and reinserted. If there was still no improvement the trachea was intubated.

The propofol and remifentanil infusions were stopped when all instruments and sheaths were removed from the joint. We removed the LMA when the patient was breathing spontaneously and muscle tone had returned to the jaw. The attending personnel in the postanaesthetic care unit were blinded to the patients’ group allocations. When the patients were able to swallow, they were given 600 mg ibuprofen PO. One hour postoperatively they were evaluated with regard to swallowing difficulties, hoarseness and sore throat on a numeric rating scale of one to ten.

The patients were discharged home with stable vital functions, absent or adequately controlled PONV and absent or adequately controlled pain. For pain control at home they were given ibuprofen (600 mg every 6 hours), metamizole (1 g every 6 hours) and tramadol as rescue medication for severe pain. A follow-up was scheduled for two days postoperatively, and airway morbidity was re-evaluated at that time.

The primary end-point of this study was the incidence of a functionally inadequate oropharyngeal seal or incorrect tip position of the LMA-S with inadequate separation of the GI and respiratory tracts. Secondary end-points were the position of the LMA-S bowl and glottic anatomy determined by endoscopy, the incidence of intraoperative ventilatory complications, the incidence and severity of airway morbidity (swallowing difficulties, sore throat, hoarseness), and the presence of blood on the cuff of the LMA-S that indicated airway lesions.

### Statistical analysis

All statistical calculations including calculations of power and sample size were performed with the program Statistica® (StatSoft Europe GmbH, Hamburg, Germany). Sample size calculations showed that two groups of 90 patients each would be required to detect a clinically relevant reduction of the incidence of an seal from the previously observed 10% to 5% with a power of 80% and a p-value of less than 0.05. To compensate for dropouts we used two groups of 95 patients each. The randomisation list was created with an online randomisation program (http://www.randomizer.org).

Continuous data were tested for normal distribution with the Kolmogorov-Smirnov test. Normally distributed data were described by mean and standard deviation, and compared by Student’s t-test for unpaired samples. Categorical data were given as absolute numbers and percentages, and analysed by Pearson’s chi-squared or Fisher’s exact test depending on the number of categories. For all tests, p < 0.05 was considered significant.

## Results

One hundred and eighty-seven patients were recruited and participated in the study. Three patients had to be excluded from final analysis due to incomplete data sets. Ninety-two patients in each group were analysed. There were 121 male and 63 female patients with an average weight of 85 ± 16 kg and an average age of 46 ± 16 years. The percentages of patients with Mallampati classes I, II and III were 51%, 36%, and 13%, respectively (Table 2).

**Table 2:** Biometric characteristics of the study population (mean (SD))

	**Control group n = 92**	**Study group n = 92**	**p**
Height (m)	1.77 (16)	1.75 (16)	
Weight (kg)	87 (16)	83 (16)	
Male/female (n)	65 / 27	56 / 36	
Age (years)	47 (16)	44 (16)	
*ASA I-III in %*			
I	58%	62%	
II	37%	34%	
III	5%	4%	
*Mallampati I-IV in %*			
I	47%	54%	
II	36%	36%	
III	17%	10%	
IV	0%	0%	
Mouth opening in cm	5.3 (1.1)	5.3 (1.1)	
Maximum thyromental distance in cm	10.5 (2.1)	10.5 (2)	

Cuff inflation did not alter the distance between the LMA fixation tab and the patient’s lip in either group indicating that inflating the cuff before securing the device did not force the it out of its original position. The two groups did not differ with regard to insertion success, oropharyngeal leak pressure, the need to use a different sized mask, or rate of failed ventilation with the LMA-S (Table 3). There was no difference in the results of the functional tests of correct position or of the fiberscopic evaluation.

**Table 3:** Study results for securing the airway (mean (SD))

	**Control group n = 92**	**Study group n = 92**	**p**
Placement success			
Success with LMA-S (%)	90%	91%	0.76
Change of LMA size (%)	20%	24%	0.47
Failure with LMA-S (%)	10%	9%	0.76
Distance fixation tab to lip			
Initial (cm)	1.5 (0.76)	1.7 (0.67)	0.11
Final (cm)	1.4 (0.62)	1.5 (0.65)	0.5
*Final LMA-S size*			0.61
3	7.6%	12%	
4	80.4%	76%	
5	12%	12%	
Position adjustment required	21.7%	16.3%	0.42
Oropharyngeal leak pressure (cmH_2_O) (mean (SD))	24.8 (6.5)	25.2 (6.2)	0.63
*Pathological clinical tests (%)*			
“Lube-tube” test	16.3%	17.6%	0.82
Sternal notch test	13%	14.3%	0.81
Gastric tube not placed	3.3%	4.4%	0.69
*Fiberscopic evaluation (%)*			
Correct position	82%	79%	0.77
Incorrect: device tip	1%	3.3%	
Incorrect: epiglottis	2%	3.3%	
Incorrect: glottis	15%	14.3%	
Glottic narrowing (n (%))	17 (19.3%)	13 (14.1%)	0.35
Of these - ventilation impaired and intervention required	13 (77%)	5 (39%)	0.04
Improved by deepening anaesthesia	4 (24%)	0 (0%)	0.06
LMA reinsertion	7 (41%)	4 (31%)	0.56
Intubation necessary	2 (12%)	1 (8%)	0.71

We observed glottic narrowing in 30 patients (17%); 13 in the study group and 17 in the control group. This caused impaired ventilation with tidal volumes of less than 6 ml.kg^-1^ in 13 of the 17 patients in the control group (77%) but in only five of the 13 patients (39%) in the study group (p = 0.04). Increasing the depth of anaesthesia was generally unsuccessful; it resolved the difficulties in four of the 13 in the control group and none of the five in the study group (p = 0.06). Two patients in the control group and one in the study group required tracheal intubation to resolve the problem. Removing and reinserting the LMA was successful in the others.

The incidence and severity of postoperative airway morbidity is shown in Table 4. There was a greater incidence of postoperative sore throat and swallowing difficulties in the control group as well as a greater incidence of blood on the cuff of the airway device.

**Table 4:** Postoperative airway morbidity

	**Control group n = 92**	**Study group n = 92**	**p**
Blood on LMA (%)	15%	5%	0.03
Dysphagia in %	30%	17%	0.04
Hoarseness in %	34%	23%	0.1
Sore throat in %	33%	19%	0.03

## Discussion

In this study we investigated the influence of the sequence of cuff inflation and device fixation on functional, anatomical and clinical parameters as well as on perioperative airway morbidity when using an LMA-Supreme®.

We were unable to confirm our hypothesis that the sequence of cuff inflation and device fixation influenced the incidence of inadequate ventilation or incorrect position of the device. Oropharyngeal leak pressures, functional tests of tip position or separation of gastrointestinal and respiratory tracts did not differ between the groups. Direct observation of the position of the device’s bowl showed similar incidences of the various types of malposition in the two groups.

We did observe a greater incidence of blood on the cuff, indicating soft tissue damage, as well as an increased rate of sore throat and dysphagia in the control group. We can only speculate whether this resulted from a greater tendency of the cuff to shift its position and abrade surrounding tissues during surgery.

The 1%, respectively 3.3% incidence of an incorrect tip position in the upper oesophageal sphincter seen through the fiberscope was much lower than the up to 40% incidence described for the classical laryngeal mask airway [10,11]. This incorrect position of the tip can facilitate pulmonary complications in non-fasting patients or when higher inspiratory pressures are required, since regurgitation and aspiration can occur, or even be provoked by gastric inflation.

Glottic narrowing occurred with equal frequency in both groups, and the observed incidence was similar to that described in previous studies [1,7,12]. However, glottic narrowing impaired ventilation significantly more often in the control group with reduced tidal volume and/or increased maximum airway pressure. This is the first description of glottic narrowing during the use of supraglottic airway devices as a possible cause of the observed ventilatory difficulties; earlier studies make no mention of any such association [1,7,12].

Glottic narrowing associated with the use of a supraglottic device is an interesting phenomenon, the cause of which is not fully understood. It seems unlikely that it is due to an inadequate depth of anaesthesia, since deepening the anaesthetic rarely improved the condition. Russo et al. suggested that the tip of the device might compress the dorsal portion of the trachea and the cricoid cartilages [7], but this does not explain why the condition does not appear immediately after insertion but only after a period with normal ventilatory parameters. One possibility is that a short period of light anaesthesia with vocal cord movement might allow the airway device to descend and “lock” the glottis in a narrowed state. This would explain why deepening the anaesthetic is not successful while removing and reinserting the airway device is. The depth of the hypnotic state was monitored by entropy in our patients and was always kept at an adequate level. However, as we have previously shown, entropy does not provide information on the adequacy of analgesia [13].

Most studies describe an incidence of airway morbidity between 8 and 19% with varying degrees of severity [3-5]. The incidences of dysphagia and sore throat were in this range in the study group (fixation followed by cuff inflation), but were significantly higher in the control group. This could possibly be because the cuff was more firmly seated and less likely to move and abrade the surrounding mucous membranes in the study group. The employed cuff inflation pressure that was at the upper limit of the manufacturer’s recommendation could have contributed to the observed high incidence of postoperative airway morbidity in the control group. In clinical practice we use a cuff pressure that is just sufficient to give a tidal volume of 6 ml kg body weight^-1^ without air leak. But since this inter-individually differing pressure would have been a confounding factor we decided to use one standard pressure for all patients. We normally use this pressure for studies [12], since it is the one most likely to allow sufficient ventilation in the greatest number of patients, but which is still within the limits set by the manufacturer.

The results of the present study apply to the LMA-Supreme® inserted as recommended by the manufacturer and blocked to a cuff pressure of 60 cmH_2_O. Any other method of inserting, affixing or otherwise using the device might, of course, affect the results in an unpredictable manner*.* The depth of intraoperative analgesia was not assessed (e.g. using the Surgical Pleth Index) and we have shown that periods of insufficient analgesia can occur even with normal depth of hypnosis in BIS or entropy [13]. This could have affected the occurrence of glottic narrowing.

## Conclusions

Changing the sequence of inflation and fixation had no effect on oesophageal leak pressure, gastrointestinal and respiratory tract separation, total incidence of glottic narrowing, rate of failed ventilation, or the incidence of incorrect mask position. Securing the position of the LMA-S before inflating the cuff reduced the incidence of airway morbidity, and of impaired ventilation due to glottic narrowing.

## Competing interests

The study was financed by departmental funds, including the purchase of all devices and materials used in the study. During the past five years none of the authors have received any form of reimbursement or financial or non-financial support from a company that could gain or lose financially from the publication of this manuscript. None of the authors hold any stocks or shares in a company that would gain or lose financially from the publication of this manuscript. None of the authors are applying for any patents related to the content of the manuscript. There are no other competing financial or non-financial interests.

## Authors’ contributions

IB initiated, designed and conducted the study, performed device insertion, analysed the data, and wrote the first manuscript draft. TAC analysed and interpreted the data and wrote the final manuscript. MR and MB were involved in study design and data analysis. HS and AM were involved in study design and were responsible for perioperative data acquisition. BB participated in study design and performed device insertions the study. JMH participated in the design of the study and was responsible for statistical analysis. All authors were funded by the department and agree to be accountable for all aspects of the work. All authors read and approved the final manuscript.

## Pre-publication history

The pre-publication history for this paper can be accessed here:

http://www.biomedcentral.com/1471-2253/14/2/prepub
